# Abattoir‐Level Beef Cattle Handling and Its Impact on Meat Quality: A Comparative Analysis of Three Abattoirs of Eastern Amhara, Ethiopia

**DOI:** 10.1155/vmi/9375068

**Published:** 2026-09-18

**Authors:** Solomon Tsegaye Adane, Ebrahim Oumer

**Affiliations:** ^1^ School of Veterinary Medicine, Woldia University, Weldiya, Amhara, Ethiopia, wldu.edu.et

**Keywords:** DFD, meat quality, pH, preslaughter handling, PSE

## Abstract

Meat quality encompasses a set of attributes preferred and evaluated by consumers based on nutritional value, conformation, sensory appeal, and safety. This research was conducted between 2024 and 2025. A cross‐sectional study was conducted using an observational study and pH evaluation of meat. Of the six welfare indicators assessed on 384 beef cattle, slipping, beating, trekking distance, and time of rest at lairage were significantly different among the abattoirs. The pH measurements of meat samples (*N* = 75) indicated that more than half (*n* = 41, 54.7%) of the total sample was found to be defective meat. Pale, soft, and exudative (PSE); red, firm, and nonexudative (RFN); and dark, firm, and dry (DFD) meat quality outcomes were significantly (*X* ^2^(4) = 11.7545; *p* = 0.019) different among the three abattoirs. The odds ratio of DFD (OR = 5.79 and 5) and PSE (OR = 7.71 and 4) meats at the Dessie and Woldia abattoirs were more than 5 and 4 times higher than at the Kombolcha abattoir, respectively. The relative risk ratio (RRR) of occurrence of PSE meat was higher for falling (RRR = 15.08; CI: 3.71–61.25; *p* < 0.001), beating (RRR = 7.33; CI: 2.01–26.76; *p* = 0.003), and slipping (RRR = 15.08; CI: 3.71–61.25; *p* < 0.001) rate increases. DFD meat was also higher on stressors such as trekking distance (RRR = 9.33; CI: 1.89–46.19; *p* = 0.006), falling (RRR = 13.26; CI: 3.61–48.64; *p* < 0.001), beating (RRR = 10.67; CI: 3.05–37.29; *p* < 0.001), and slipping (RRR = 10.88; CI: 3.02–39.09; *p* < 0.001). In contrast, intermediate lairage stay was not found to be statistically significant for the development of DFD meat (RRR = 13.26; CI: 3.61–48.64; *p* = 0.270). Therefore, this study concluded that preslaughter handling and meat quality outcomes varied across export and municipal setting abattoirs.

## 1. Introduction

### 1.1. Background and Justification

Meat quality could be altered due to several factors, such as preslaughter animal handling, the hygienic status and facilities of the abattoir, and postslaughter contamination. Improper animal handling and lack of appropriate and functional facilities result in heavy stress on beef cattle that compromises the welfare status and ultimately affects the meat quality by initiating muscle glycogenolysis. Glycogenolysis, on the other hand, increases the acidification of muscle during slaughter, and the result will be pale, soft, and exudative (PSE) meat [1].

The public nowadays is demanding meat produced following good animal preslaughter handling on the basis of animal welfare standards [2]. Many studies have been done in Ethiopia on preslaughter handling and transportation effects on meat quality [3–5].

All raised public abattoir challenges in Ethiopia have a direct impact on beef cattle welfare and ultimately on meat quality. Welfare of animals is compromised during preslaughter handling that induces stress and consequently deviation from meat quality standards [6]. So, measuring welfare parameters could assist in evaluating the quality of meat associated with animal handling.

Studies conducted abroad showed that poor preslaughter handling of animals could negatively affect meat quality [1, 6–10]. Similarly, many studies in Ethiopia indicated poor abattoir‐level animal handling, which resulted in poor meat quality outcomes [3, 5, 11–14]; however, none of these studies included antemortem indicators, a comparison between municipal and export standard abattoirs and their association with different meat quality outcomes. Therefore, the objective of this study was to assess the effect of preslaughter handling on meat quality outcomes.

## 2. Materials and Methods

### 2.1. Description of Study Area

The Kombolcha ELFORA export standard abattoir setting, which is found in the South Wollo Zone of the Amhara National Regional State, Ethiopia. Kombolcha lies between 11°5′ and 11°50′ N latitude and 39°44′ E longitude. The elevation is between 1842 and 1915 m.a.s.l. The abattoir is a private agroindustrial company with a daily cattle slaughter capacity of 250–300 [15].

The Dessie municipality abattoir is located in Dessie city, the South Wollo zone of the Amhara National Regional State, Ethiopia. The city lies between 11°8′ N latitude and 39°38′ E longitude, with an elevation between 2470 and 2550 m.a.s.l. The average rainfall ranges from 851.3 to 1612.6 mm. The abattoir has a daily cattle slaughter capacity of 35–40 [16].

The Woldia municipality abattoir is located in Woldia City, the North Wollo Zone of the Amhara National Regional State, Ethiopia. Woldia lies between 11°48′ and 11°50′ N latitude and 39°34′ and 39°36′ E longitude. The elevation is between 1379 and 2400 m.a.s.l. The abattoir has a daily capacity of slaughtering 35–40 cattle [17].

### 2.2. Study Design

The cross‐sectional study method was used together with observational welfare indicator measurements and meat pH measurements to study the preslaughter handling method, the welfare status of the animals, and meat quality tests.

### 2.3. Sample Size Determination

For preslaughter handling assessment, sample size was calculated based on 50% prevalence, which was 384 cattle, determined by using a formula developed by Cochran [18].

(1) N=z2×p×qe2=384,

where *N* is the sample size for the study, *Z* is linked to a 95% confidence interval (1.96), and *p* is the estimated proportion of an attribute that is present in the population, which is 0.50 in this study. *q* = 1−*p*, that is, 0.50, and *e* is the desired level of precision, which is 0.05.

For meat pH measurement, a total of 75 meat samples were taken from three abattoirs, with 25 samples from each.

### 2.4. Data Collection

Data were collected using direct observation of antemortem welfare indicators and meat pH measurements. Direct observation and a checklist were used, including the transportation system, origin of the beef cattle, lairage stay time, and handling by abattoir attendants. Signs of poor handling, such as vocalization, beating, falling (all four legs losing contact), and slipping (loss of footing), were recorded.

#### 2.4.1. Study Animals

A total of 384 cattle were examined, most of which were primarily local zebu breed, but there were also 23 Holstein‐Friesian crossbreeds. All cattle were male, with the average live body weight ranging from 180 to 300 kg.

#### 2.4.2. Observational Study at Lairage

Observations on time of rest, water availability, and falling/slipping/vocalization/beating frequency were recorded. The lairage stay was recorded as < 3, 3–12, and > 12 h based on the standard mentioned by Yu et al. [19]. Slipping, falling, and vocalization were the physical indicators of improper handling, such as pushing, overcrowding, and stress due to pain from slaughtering. The observation of slipping, falling, and vocalization was performed in the abattoirs, specifically at the lairage, stunning, and slaughtering area. The evaluation of slipping, falling, and vocalization was performed based on the standards set by the European Food Safety Authority [20].

#### 2.4.3. Antemortem Welfare Measurement

During the observational study, animal welfare indicators were evaluated and recorded as per the recommendations set by [21]. The procedures were as follows:

Welfare measurement was done based on antemortem welfare indicators [22]. The indicators used before slaughter were the presence of slipping, beating, falling, and vocalization. When there was a total loss of contact of all legs, it was counted as falling, and slipping was counted when there was any loss in footing other than all legs. Vocalization as an indicator was only considered when there was a hit by handlers and other types of mishandling of beef cattle.

#### 2.4.4. Determination of Meat Quality Based on the pH Value

The pH_24 hours_ value of meat was measured using a digital pH meter (MP511, China). During sample collection, meat samples were collected from the longissimus dorsi at the 10th rib, and the samples were immediately transported to the Woldia University biology laboratory for refrigeration at 3°C for 24 h. After 24 h, pH_24 hours_ was measured using a calibrated digital pH meter following the procedure stated by the European Food Safety Authority [20]. The pH meter was first calibrated using pH buffers (pH 4.0 and 7.0) and adjusted for temperature. Rinsing of the electrode using distilled water was always done before and after each measurement, and then meat was categorized into three groups: red, firm, and nonexudative (RFN/Normal; pH: 5.4–6.0); dark, firm, and dry (DFD; pH: > 6.0); and PSE (pH: < 5.4) [23, 24].

### 2.5. Data Analysis

Data were entered into Excel and then analyzed using STATA 15.0. Descriptive statistics were used to analyze preslaughter handling and welfare‐related outcomes. A chi‐square test was used to see if stressors were present and significantly affected meat quality and varied across abattoirs. A *p* value of less than 0.05 was considered significant. Multinomial logistic regression was used for the comparison of abattoirs on DFD and PSE meat. The relative risk ratio (RRR) was also used to compute the effect of welfare indicators on meat quality outcomes.

## 3. Results

### 3.1. Welfare Indicators and Their Effect on Cattle Welfare

A total of six welfare indicators were assessed across three abattoirs, of which three were animal‐based and the other three were management‐based factors. Animal‐based welfare indicator measurements such as vocalization (*p* = 0.065) and falling (*p* = 0.091) showed no significant difference among the abattoirs, whereas slipping (*p* = 0.033) showed a significant difference between abattoirs. Management‐related factors such as beating, lairage rest time, and trekking distance showed a statistical difference (*p* < 0.001) between the three abattoirs. The vast majority of beef cattle (86.9%) in the Kombolcha ELFORA abattoir rested for more than 12 h (*n* = 146), while in both the Woldia and Dessie abattoirs, 95.3% and 91% of beef cattle rested in the lairage for less than 3 h, respectively (Table 1).

**TABLE 1:** Welfare indicators and their status across the three abattoirs.

Key welfare indicators	Yes/no	Frequencies (percentage) across three abattoirs			
		Kombolcha ELFORA	Dessie	Woldia	Chi‐square test (*p* value)
Falling	Yes	17 (10.1%)	21 (17.2%)	17 (19.5%)	*p* = 0.091
	No	151	101	70	

Slipping	Yes	12 (7.1%)	17 (13.3%)	10 (11.5%)	*p* = 0.033
	No	156	111	77	

Vocalization	Yes	61 (36.3%)	47 (36.7%)	33 (37.9%)	*p* = 0.065
	No	107	81	54	

Beating	Yes	35 (20.8%)	77 (60.2%)	18 (20.7%)	*p* = 0.000
	No	133	51	69	

Trekking distance	< 30 km	8 (4.76%)	26 (20.3%)	12 (13.48%)	*p* = 0.000
	> 30 km	160 (95.24%)	102 (79.69%)	75 (84.3%)	

Time of rest at lairage	< 3 h	6 (3.6%)	122 (95.3%)	81 (91.01)	*p* = 0.000
	3–12 h	16 (9.5%)	2 (1.56)	3 (3.37%)	
	> 12 h	146 (86.9)	4 (3.13%)	5 (5.62%)	

### 3.2. Meat Quality Based on pH Value of Meat

A frequency analysis conducted (Table 2) on meat quality categories, which were PSE, RFN, and DFD meat, indicated that half (*n* = 41, 54.7%) of the total sample (*N* = 75) was found as either PSE or DFD meat. The highest frequency of abnormality was DFD meat (*n* = 23, 30.67%), followed by PSE meat (*n* = 18, 24%) (Table 3).

**TABLE 2:** Comparison of meat quality across abattoirs.

Abattoir	PSE	RFN	DFD	Total
Kombolcha ELFORA	3 (12%)	18 (72%)	4 (16%)	25 (100%)
Dessie abattoir	9 (36%)	7 (28%)	9 (36%)	25 (100%)
Woldia abattoir	6 (24%)	9 (36%)	10 (40%)	25 (100%)
Total	18 (24%)	34 (45.33%)	23 (30.67%)	75 (100%)

*Note:* *X* ^2^(4) = 11.7545 *p* value = 0.019.

**TABLE 3:** Frequencies of meat quality outcomes.

Meat quality	Frequency	Percent	Cumulative percentage
PSE	18	24%	24.00
RFN	34	45.33%	69.33
DFD	23	30.67%	100.00
Total	75	100	

A chi‐square test was conducted to see the relationship between meat quality categories and abattoirs. The result in Table 4 indicated that meat quality characteristics had a statistically significant difference among the three abattoirs (*X* ^2^(4) = 11.7545; *p* value = 0.019).

**TABLE 4:** Abattoir comparison of DFD and PSE meat using logistic regression.

Meat quality	Variable	Abattoirs		
		Kombolcha ELFORA	Dessie abattoir	Woldia abattoir
DFD meat	Odds Ratio (OR)	0.22	5.79	5
	95% Confidence Interval (CI)	0.75–0.66	1.34–25.06	1.22–20.46
	*p* value	0.007	0.019	0.025

Overall chi‐square [prob > chi2]		0.0202		

PSE meat	Odds Ratio (OR)	0.167	7.71	4
	95% Confidence Interval (CI)	0.049–0.566	1.60–37.13	0.81–19.82
	*p* value	0.004	0.011	0.09

Overall chi‐square [prob > chi2]		0.0209		

Multinomial logistic regression was used for the comparison of abattoirs on DFD and PSE meat (Table 5), which indicated that there was a significant statistical difference in both PSE meat (overall *p* = 0.0209) and DFD meat (overall *p* = 0.0202) between the abattoirs. The Odds Ratio (OR) regarding DFD meat showed that DFD meat in the Dessie and Woldia abattoirs was 5 times (OR = 5.79 and 5) higher than that of the Kombolcha ELFORA abattoir. The OR measurement for PSE meat indicated that PSE meat in the Dessie abattoir was 7.7 times higher than in the Kombolcha ELFORA abattoir. Similarly, PSE meat in the Woldia abattoir was 4 times higher than that of the Komolcha ELFORA abattoir.

**TABLE 5:** The association of preslaughter welfare indicators with meat quality outcomes.

Welfare indicators		Meat quality outcomes					
		PSE			DFD		
		RRR	*p* > |*z*|	95% CI	RRR	*p* > |*z*|	95% CI
Trekking distance	> 30 km	1.40	0.573	0.44–4.47	9.33	0.006	1.89–46.19
	_cons	0.44	0.068	0.18–1.06	0.13	0.006	0.03–0.54

Lairage stay	3–12 h	0.43	0.373	0.07–2.76	0.35	0.270	0.06–2.25
	> 12 h	0.09	0.005	0.02–0.49	0.16	0.006	0.04–0.58
	_cons	1.17	0.695	0.54–2.52	1.42	0.356	0.68–2.97

Beating	Present	7.33	0.003	2.01–26.76	10.67	0.000	3.05–37.29
	_cons	0.25	0.001	0.11–0.57	0.25	0.001	0.11–0.57

Falling	Present	15.08	0.000	3.71–61.25	13.26	0.000	3.61–48.64
	_cons	0.17	0.000	0.07–0.45	0.24	0.001	0.11–0.55

Slipping	Present	15.08	0.000	3.71–61.25	10.88	0.000	3.02–39.09
	_cons	0.17	0.000	0.13–0.60	0.28	0.001	0.13–0.60

PSE meat, as indicated in Table 5, showed a higher association with acute stressors in increasing the RRR of occurrence, such as beating (RRR = 7.33; CI: 2.01–26.76; *p* = 0.003), falling (RRR = 15.08; CI: 3.71–61.25; *p* < 0.001), and slipping (RRR = 15.08; CI: 3.71–61.25; *p* < 0.001). Extended lairage stay (> 12 h) also showed statistical significance in the development of PSE meat (RRR = 0.09; CI: 0.02–0.49; *p* = 0.005). On the other hand, the impact of long‐distance trekking (> 30 km) was not statistically significant on PSE meat occurrence.

The development of DFD meat was significantly associated with both chronic and acute stressors, such as trekking distance > 30 km (RRR = 9.33; CI: 1.89–46.19; *p* = 0.006), beating (RRR = 10.67; CI: 3.05–37.29; *p* < 0.001), falling (RRR = 13.26; CI: 3.61–48.64; *p* < 0.001), and slipping (RRR = 10.88; CI: 3.02–39.09; *p* < 0.001). In contrast, intermediate lairage stay was not found to be a statistically significant factor for the development of DFD meat (RRR = 13.26; CI: 3.61–48.64; *p* = 0.270) (Table 5).

## 4. Discussion

Welfare indicators such as rest after transportation and behavioral indicators like vocalization, falling, and slipping were critical factors that can lead to poor meat quality [8]. Assessment of animal welfare at abattoirs uses animal‐based measures, resource‐based indicators, and animal handling protocols [25].

This research investigated the welfare status of animals before slaughter at the abattoir level. The study indicated that there were no statistical differences in falling and vocalization between the abattoirs. The rate of falling was between 10% and 19.5%, and vocalization was 36%–37.9% of the slaughter cattle. This finding was comparable with findings of Frimpong [26] at the Kumasi abattoir, Ghana, where high rates of falling (52.5%) and vocalization (14%) were recorded. The absence of variation in these two animal‐based welfare indicators between municipality abattoirs and export abattoirs indicated that there was malpractice during animal handling.

Unlike falling and vocalization, animal‐based welfare indicators, slipping showed a significant difference (*p* = 0.033) between the three abattoirs. The Kombolcha ELFORA counted the highest rate of slipping, while the Dessie and Woldia abattoirs counted nearly equal rates of slipping. The reason behind this could be floor infrastructure and lairage crowding, resulting in fights between cattle, consequently resulting in slipping [27]. A similar study [8] at Korean cattle slaughterhouses indicated that the slipping rate was between 10% and 30% at six different abattoirs with a statistically significant difference (*p* < 0.001). The Dessie abattoir recorded the highest slipping rate. As Grandin [27] suggested, to maintain optimal welfare, the percentage of slipping should be below 3%. Based on this benchmark, all three abattoirs failed to fulfill the standard limit.

To minimize the high rate of slipping and falling, there should be targeted interventions focusing on lowering stress by minimizing the number of animals at the lairage, physical facility improvements, and restructuring the floor to be nonslippery [27, 28]. A higher rate of vocalization indicated the compromised welfare status of cattle due to fighting each other, overcrowding at the lairage, aggressive handling and beating by attendants, and an inappropriate stunning method with repeated stabbing using a sharp knife by unskilled abattoir workers. Facility improvement is essential to avoid stress due to crowding at the lairage; repeated stunning should be replaced with other stunning methods. Furthermore, continuous training of abattoir personnel will improve the welfare status [8, 27].

There was a significant difference (*p* < 0.001) between abattoirs in beating rate. The beating rate was very high at the Dessie abattoir, while the Kombolcha ELFORA and Woldia abattoirs were similar. The incidence of beating (60.2%) in the Dessie abattoir was higher than the report at the Jimma abattoir, which was 8.9% [29] and lower than the 87.7% reported by Bekele Atoma et al. [30]. This variation could be due to a lack of training on animal behavior and standard operating procedures in most municipal abattoirs [5]. The high rate of beating is a welfare concern that consequently results in economic loss due to carcass condemnation of bruised meat, as stated by Gadisa Muleta [31].

In this study, trekking was the dominant mode of transportation, with 88% of cattle moved long distances (> 30 km). This indicated higher welfare concerns associated with high physical stress and dehydration, which increases the incidence of DFD meat. The reliance on long‐distance trekking without adequate rest was also common practice in other parts of Ethiopia. This finding was consistent with the studies done in Dendi and Welmara districts [5] and in Guder and Ambo abattoirs [3], where 55.9% and 72% of beef cattle were transported to the abattoir using trekking, respectively.

More than 90% of beef cattle at the Dessie and Woldia abattoirs stayed in lairage for a very short time (< 3 h), while most beef cattle (86.9%) brought to Kombolcha ELFORA stayed for more than 12 h. In Dessie and Woldia, beef cattle coming after long‐distance trekking were slaughtered within 3 h of staying in lairage, which might be the major factor in low performance in meat quality. According to Davis et al. [9], adequate rest is essential for preslaughter recovery; otherwise, it leads to DFD meat that results in poor meat quality.

The shortest resting time of beef cattle at these abattoirs, specifically at Dessie and Woldia, could be the main reason for the poor quality of meat. Yu et al. [19] described that lairage time should be between 3 and 12 h. Both short stays (< 3 h) and longer stays (> 12 h) have their own negative effect on meat quality. The shortest lairage stay was similar to other studies conducted previously, such as Bekuma and Tadesse [5] in Dendi and Walmera abattoirs and Diro et al. [3] in Ambo and Guder abattoirs, with the result of 0–3 h of stabilization time after stressful long‐distance trekking, and Gadisa Muleta [31] observed that 100% of beef cattle lairage stays were less than 3 h at the Haramaya University abattoir. The shortest duration of lairage stay affects meat quality through stress response, especially after long transportation, and a longer duration of stay requires water and feed; otherwise, it has a negative effect on meat quality [19].

After the assessment of preslaughter factors, this study conducted measurements of the pH of meat and found that more than half of the meat samples taken had quality defects, either as DFD or PSE meat. DFD had a higher rate of recorded meat quality defects than PSE. The higher rate of DFD meat could be due to stress related to long‐distance trekking and short lairage rest time. This finding was comparable to the finding in Ambo and Gudar abattoirs, where a 47% rate of DFD meat was reported [3]. The same high rate (47%) of DFD meat was also reported in Dendi and Walmera abattoirs [5]. Studies conducted in Mexico and Ghana found a high rate of DFD meat, which was 90.4% [7] and 86% [26], respectively. The reason for the high rate of DFD meat in those previous studies was long‐distance transportation and short time for rest before slaughter.

A chi‐square test and multinomial logistic regression showed that meat quality outcomes were significantly different among the three abattoirs. DFD meat at the Dessie and Woldia abattoirs was 5 times higher than the rate of DFD meat in the Kombolcha ELFORA abattoir. The reason behind this could be the lairage resting time, where both the Dessie and Woldia abattoirs had 10 h less rest time than the Kombolcha ELFORA abattoir. The long‐distance trekking combined with short resting time could be the main reason resulting in a high rate of DFD meat, which was also supported by other previous studies done in Dendi and Walmera abattoirs [5], in the Ethiopian livestock chain [32] and in China [33].

Comparing the three abattoirs on the occurrence of PSE meat showed that the rate of PSE meat at the Dessie abattoir was 7.7 times higher than the PSE meat found at the Kombolcha ELFORA abattoir. Similarly, the rate was also 4 times higher at the Woldia abattoir compared to the rate of PSE at the Kombolcha ELFORA abattoir. PSE is mostly associated with acute short‐term stressors such as beating, slipping, and falling [27, 34, 35]. The Dessie abattoir showed a higher rate of PSE meat, followed by the Woldia abattoir. The rate of PSE in this study was higher than the 4% PSE meat found at the Ambo and Guder abattoirs [3] and 5.6% of PSE meat found at the Dendi and Welmera abattoirs [5]. The difference could be due to the preslaughter handling of beef cattle at the abattoir level.

In this study, acute stressors, such as beating, falling, and slipping, were the major causes of the development of PSE meat. This finding was supported by previous studies [27, 34, 35]. But, on the other hand, DFD meat outcomes were associated with both chronic and acute stressors such as long‐distance trekking, beating, falling, and slipping. A study in Mexico [7] indicated that chronic stressors and acute handling failures (bruises and injuries) at the abattoirs were the main reason for the development of DFD meat. Studies indicated that both chronic and acute stressors could result in DFD meat, such as studies by Bekuma and Tadesse [5] and Carrasco–Garcia et al. [7], which listed long‐distance trekking without sufficient rest as a primary determinant of DFD meat, while Davis et al. [36] explicitly demonstrated that acute stressors led to DFD meat.

During the study period, trekking distance as a stressor was highly associated with DFD meat, with long distance having a nine‐fold RRR. This finding was supported by previous research [5, 7, 36]. Lairage resting time > 12 h was having a significant association with DFD meat. The reason behind it might be associated with the exhaustion of energy with added prolonged exposure to stressors such as prolonged fasting, fighting, and overcrowding. Lee [37] confirmed that extended lairage time over 12 h significantly increases the incidence of DFD meat. In addition, studying the effect of different lairage stays on meat quality characteristics by Yu et al. [19] indicated extended stays at lairage could introduce new stressors such as social frictions and behavioral distress, which result in the development of DFD meat.

The RRR of beating (7.33, 10.67), falling (15.08, 13.26), and slipping (15.08, 10.88) was high for both PSE and DFD meat, respectively. The RRR indicates that these three factors were the predominant stressors with significant associations (*p* < 0.05) with meat quality outcomes. While acute stressors are typically a direct cause for PSE meat, the high association of DFD meat with the acute stressors could be due to prolonged abuse during loading, transporting, or lairage stay, in which the justification was in agreement with the explanation of Song and Chun [8, 38], where both stated that based on time reference, both PSE and DFD meat might be the result of the same factors, like beating.

## 5. Conclusion

The research showed huge disparities between the three abattoirs in almost all parameters studied. The export standard abattoirs and municipality abattoirs in Eastern Amhara revealed significant differences in preslaughter handling and the rate of defective meat quality outcomes. The Kombolcha ELFORA abattoir relatively maintained the standard handling of animals prior to slaughter compared to the Dessie and Woldia abattoirs. Beef cattle welfare was highly compromised in municipality settings by physical abuse, lack of water, long‐distance transportation, mainly trekking, and short resting time. Regarding meat quality outcomes, more than 70% of the samples from the Kombolcha ELFORA abattoir were found to have good‐quality meat. Conversely, over 60% of the samples from the Dessie and Woldia abattoirs exhibited either PSE or DFD meat characteristics, indicating major meat quality defects at municipal abattoir settings. Generally, the municipal abattoirs had major limitations in preslaughter handling and standard practices, which in turn negatively affected the meat quality produced.

**Nomenclature**
DFD: Dark, firm, and dry
OR: Odds ratio
PSE: Pale, soft, and exudative
RFN: Red, firm, and nonexudative
RRR: Relative risk ratio

## Author Contributions

Solomon Tsegaye Adane conceived and proposed, collected data, proofread, and edited the data, analyzed and wrote up the final article.

Ebrahim Oumer collected data, wrote the final article, and reviewed and corrected the article.

## Funding

This study did not receive any funds but is a part of Woldia University’s thematic research, Ethiopia.

## Ethics Statement

The Ethical Review Board of Woldia University [reference number: WDU/IRB/003/2024; approval date: July 18, 2024 G.C.] evaluated and approved the study. As this research did not involve live animal handling or euthanasia, the ethical review committee confirmed that the research uses observation, so it has no ethical concerns. Additionally, we secured verbal consent from the abattoir to access the abattoir to investigate.

## Consent

Please see the Ethics statement.

## Conflicts of Interest

The authors declare no conflicts of interest.

## Data Availability

Data are available on request due to privacy/ethical restrictions.
